# Adventitious Root Formation in Plants: The Implication of Hydrogen Peroxide and Nitric Oxide †

**DOI:** 10.3390/antiox12040862

**Published:** 2023-04-02

**Authors:** Peter Anargyrou Roussos

**Affiliations:** Laboratory of Pomology, Department of Crop Science, Agricultural University of Athens, Iera Odos 75, 118 55 Athens, Greece; roussosp@aua.gr; Tel.: +30-210-529-4596

**Keywords:** auxins, cuttings, reactive oxygen species, reactive nitrogen species, rhizogenesis, signaling

## Abstract

Adventitious root formation is defined as the formation of new roots on above-ground plant parts and is considered crucial for the survival of a plant under harsh environmental conditions (i.e., flooding, salt stress, and other abiotic stresses) as well as in the nursery industry. Clonal propagation is based on the ability of a plant part to grow and generate a completely new plant, genetically identical to the mother plant, where the plant part came from. Nurseries exploit this potential by multiplying millions of new plants. Most nurseries use cuttings to achieve that, through the induction of adventitious root formation. Many factors have been implicated in the capacity of a cutting to root, with the major role being played by auxins. During the last few decades, intense interest has emerged in the role of other potential rooting co-factors, such as carbohydrates, phenolics, polyamines, and other plant growth regulators, as well as signal molecules, such as reactive oxygen and nitrogen species. Among the latter, hydrogen peroxide and nitric oxide have been found to play significant roles in adventitious root formation. Their production, action, and general implication in rhizogenesis are discussed in this review, in terms of interaction with other molecules and signaling.

## 1. Introduction

**Figure 1:**
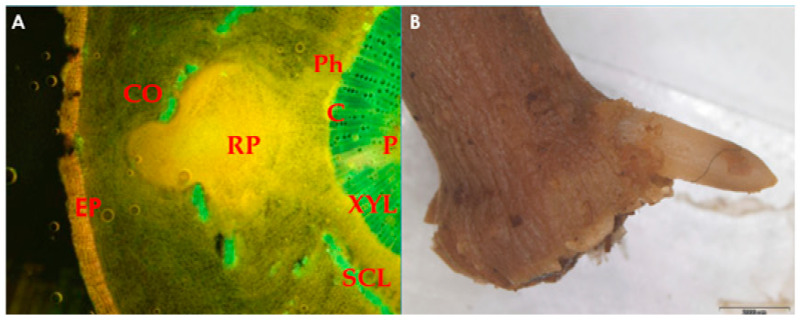
(**A**) Microscopic image of root primordium (RP) development in olive leafy cutting, originating from the area around the cambium (C), which disrupts the continuum of the sclerenchymatous ring (SCL) and passes through the cortex (CO); (**B**) root emergence at the base of olive sub-apical shoot cutting. EP, epidermis; XYL, xylem; P, pith; Ph, phloem. The photo is courtesy of Prof. Nikoleta-Kleio Denaxa.

A plant root system is composed of the primary roots, mainly formed at the embryogenic and later seedling stage, the lateral and the adventitious roots (ARs) [1,2,3]. The latter are mainly formed on above-ground plant parts [4] when the conditions are favoring their formation and development and are considered post-embryogenic roots [5]. Organs, where ARs can be formed, are shoots, leaves, stems, nodes, and hypocotyls, while underground stems and old root parts can also be the source of ARs [3,5,6]. In contrast, lateral roots (LRs) are formed on primary roots [7]. Another major difference between ARs and LRs is their origin, as LRs come from the pericycle cells, while the ARs originate from regions around the cambial zone or other meristematic tissues (Figure 1) [4,6,7]. Both ARs and LRs present similar properties, as both serve for nutrient and water uptake, while they are also sources of endogenous cytokinin production. On the other hand, the formation of ARs is mostly observed under harsh stress conditions, such as flooding or hypoxia, after mechanical wounding or in the case of vegetative plant propagation where the main objective is the formation of ARs on plant parts and organs (propagules) in order to produce a new line of plants [8,9,10,11]. In clonal propagation nowadays, the most commonly used propagation material is the cutting. In general, every plant part, which can be severed from the mother plant and, under favorable conditions, can regenerate the plant it originates from, can be a potential cutting. Many organs can be used as cuttings, from a simple leaf, cut into pieces, to a stem with or without leaves, a single bud, a root cutting, an explant in in vitro propagation, and other types of propagules [12].

**Figure 2:**
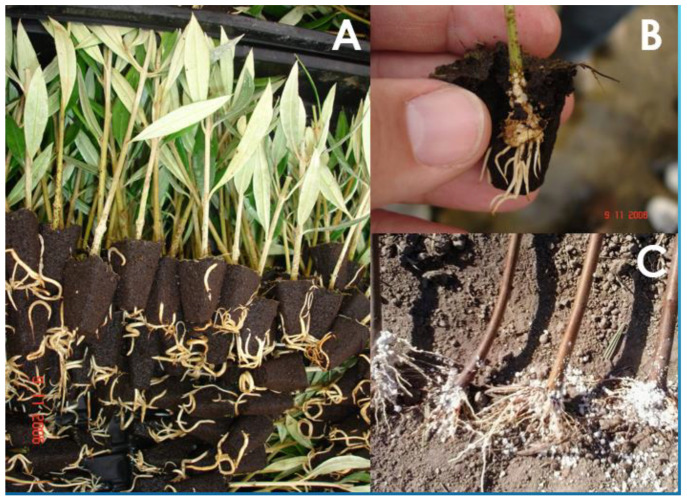
Rooted olive softwood cuttings (**A**), details of the root system emergence (**B**), and (**C**) rooted hardwood cuttings of Rootpac-90 peach rootstock.

Adventitious root formation (ARF) in cuttings is a prerequisite step in the clonal propagation of economically important plant species, taking place in nurseries multiplying horticultural, ornamental, forestry, or medicinal plants (Figure 2) [4,9,10,13]. The aim of ARF is the production of a perfect, complete, and autonomous plant deriving from a cutting. For the nursery to be successful, the losses during, as well as after, the ARF should be minimized. Such losses are usually due to either insufficient rooting performance or poor quality of the rooting system produced, which presents a barrier to the successful acclimatization of the rooted plantlets [9,14,15].

**Figure 3:**
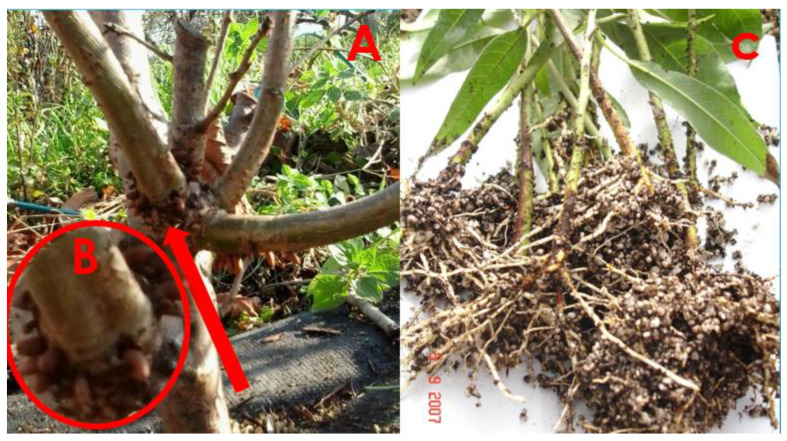
Pre-formed root initials in cherry rootstock COLT (**A**) with a close look at their emergence (**B**) and wound plus auxin-induced roots in peach-almond rootstock GF677 softwood cuttings (**C**).

Stem cuttings are the most popular among the organs, which can be used in the vegetative propagation of plants (i.e., leaves, roots, buds, and other plant parts able to regenerate the mother plant) [12]. There are two ways ARF can occur in stem cuttings: via preformed root initials and through wound-induced rooting (Figure 3) [16]. The pre-formed or latent root initials already exist in the cut stem and lay dormant by the beginning of stem development [16]. They are activated through the severance of cutting from the mother plant, and under favorable environmental conditions, they can develop into functional roots [16]. On the other hand, wound-induced ARF is the major type of rhizogenesis taking place in stem cuttings [16]. For the ARF to be efficient, two major factors are crucial, i.e., the presence of the necessary auxin (either the endogenous and/or the applied one), and a tissue predisposed to form roots, as it is known that there is a great difference between juvenile and mature tissues in their ability to form roots, deriving even from the same plant source [16,17]. Besides these two, many other factors are essential, to achieve high rooting percentages, either endogenous or exogenous (hormonal balance, water relationships, oxygen, relative humidity, light, pathogens, and others) [12]. High rooting percentages during asexual plant propagation are the aim of all nurseries, along with short rooting period, high root number, dense rooting system, medium root length (not too short but also not too long roots, which are quite fragile during transplanting), which are all considered as good markers for a successful rooting.

Nonetheless, insufficient rooting still occurs, and economic losses are faced despite the optimized environmental conditions, the state-of-the-art equipment, and the careful organization and management of the production line in the modern propagation industry. A deeper and more holistic understanding of the physiological, biochemical, and molecular mechanisms of ARF is therefore essential for improving the existing propagation protocols.

## 2. Insights into Adventitious Root Formation and Factors Affecting It

ARF is usually divided into various phases, based on physiological, biochemical, and molecular markers. De Klerk et al. [14] have described these phases starting from the dedifferentiation of already differentiated cells at the base of the cuttings and their reprogramming, which are usually found in the vicinity of the vascular cambium, secondary phloem, and surrounding tissues. In poplar, the adventitious roots seem to originate from the area between the phloem and cambium [18], while in apple, the interfascicular cambium cells are responsible for the origin of adventitious roots [18]. In raspberry, on the other hand, the ray cells are those to be dedifferentiated and become the progenitor cells for the root primordium formation [18]. These cells should then become competent to respond to the rooting stimulus, which in most cases is being played by auxin, thus promoting the formation of root meristemoids (for some researchers, this is considered the early induction phase) [6,16]. During the next phase (the induction or late induction phase), these cells become committed to forming the first root initials [9,16]. Right after, a series of cell divisions occur, where the organized mass of cells is visible, forming the first root meristem, which will gradually develop into root primordium (root initiation) [4,9,19,20,21]. The latter will continue to grow in order to connect to the vascular system of the cutting [7] while at the same time, if the conditions are favorable, it will emerge from the stem as a functional, developing root (expression phase or root emergence) [4,18].

Although this description of the phases of ARF seems like a simplistic approach and a simple procedure, it is far from that, as it is a complex phenomenon regulated by both endogenous and exogenous factors.

Once the cutting is separated from the donor plant, the soil–plant–atmosphere continuum is interrupted [9]. The breakdown of the vascular continuum then induces an accumulation of auxin near the wounding zone, within the first hours after excision [6,9,22,23]. Wound-responsive genes activate all the necessary mechanisms of wound sealing and pathogen attack prevention [9]. At the same time, the basipetal polar auxin transport (PAT) is triggered (from the site(s) of synthesis, i.e., the shoot apex and young leaves, to the stem base), and auxin transporters take control and induce the canalization of auxin to the cutting’s base, which results in the prementioned accumulation of auxin around the basal zone [6,10]. This auxin accumulation, along with the potent exogenous supply, may trigger the series of phases described above. Along with this triggering, auxin also induces an accumulation of soluble sugars (mainly in the form of sucrose) [24], which will be primarily used as carbon skeleton donors and energy sources for the sealing of the wound and the initial stages of adventitious roots [9,25,26]. Thus, an auxin-carbohydrate cross-talk takes place during the initial stages, establishing a new carbohydrates sink at the base of the cutting [26]. During that time, the application of auxin seems to accelerate the initial biochemical changes observed at the base of the cuttings, as for some easy-to-root species, this auxin application may not be necessary.

It should be noted, however, that while a transient increase in the levels of auxin is needed during the induction phase, this is followed by a gradient decrease during the initiation and expression phases (even though increases in auxin levels have been detected during the expression phase) [26,27,28]. Overall, auxin concentration within the cutting is controlled through synthesis and degradation, transportation, and conjugation or de-conjugation, as regulatory mechanisms to adjust its desired level [29].

During the induction phase, apart from auxin and carbohydrate accumulation, several other biochemical changes take place, some of which include the local increase in jasmonate as well as of some phenolic compounds, reactive oxygen species (ROS), changes in the levels of other plant growth regulators (especially an increase in ethylene production due to wounding) and changes in the activity of enzymes such as peroxidases (PODs), phenoloxidases, and others [10,13,22,23]. Peroxidases are heme-containing enzymes with multifunctional roles and various organic substrates [30], including the endogenous auxin indole-3-acetic acid (IAA), and this is the reason for which they are considered classical rooting markers [10,11,31,32]. Polyamines have also been implicated in the rooting process since they have been found to promote ARF in some species and improve their response to the external application of auxin [11,16,33,34,35,36]. All classes of phytohormones (cytokinins, ethylene, abscisic acid, and gibberellins) have also been implicated (positively, negatively, or neutral) in the capacity of a cutting to form roots [6,13,22,37,38].

Apart from the prementioned organic molecules, nutrients also play an important role in cutting behavior during ARF, as structural elements, enzyme co-factors, and signaling molecules [1,10,39,40,41]. All the above-mentioned organic and inorganic constituents of a cutting may act either as the co-factors or inhibitors of ARF, which largely depends on the genetic material and their concentration changes during ARF as well as their ratios. It has been shown that the ease of ARF is a quantitative heritable trait, which is under the influence of many factors [42].

The actual list of environmental and endogenous factors that affect ARF includes nearly every factor that can affect plant growth and development. Among these, the following have been recognized as potent ARF regulators:

- Traditional plant growth regulators (auxins, cytokinins, gibberellins, abscisic acid, and ethylene);
- Light intensity, quality, and photoperiod;
- Oxygen and carbon dioxide levels;
- Free radicals;
- Relative air and soil humidity;
- The pH and physical properties of the substrate;
- Antioxidants;
- Polyamines;
- Nutrients;
- Specific growth regulators such as strigolactones, jasmonates, brassinosteroids, melatonin, and generally indoleamines and catecholamines;
- Hydrogen;
- Hydrogen sulfide;
- Methane;
- Calmodulin;
- Salicylic acid;
- Amino acids;
- Mitogen-activated protein kinase (MAPK);
- Ca^2+^-dependent protein kinase (CDPK);
- Cyclic guanosine monophosphate (cGMP) and others [1,32,36,43,44].

It becomes obvious then that ARF is anything but simple, with an array of factors interacting with each other, regulating its outcome. However, in recent years, and by exploiting the potential of instrumental analysis (both at the biochemical and molecular level), several novel signal molecules have been identified as potential regulators of ARF. Among those, ROS, as well as the reactive nitrogen species (RNS), have been recognized as important players in both lateral root formation as well as ARF [3,45,46]. Among these, hydrogen peroxide (H_2_O_2_) and nitric oxide (NO) have been implicated as modulators of induction and initiation phases.

## 3. Oxidative Species (OS) and Their Role

Reactive oxygen species are products of aerobic metabolism. They are produced in all living organisms, and under normal growth and developmental conditions, their production and scavenging are efficiently balanced. The incomplete reduction of molecular oxygen is the generative force for their production. The most important ROS are hydrogen peroxide (H_2_O_2_), superoxide anion (O_2_^•−^), hydroxyl radicals (^•^OH), singlet oxygen (^1^O_2_), and others [47,48]. Mitochondria, peroxisomes, and chloroplasts are all cellular organelles where the production of ROS occurs [48], while an apoplastic oxidative burst also confers to the accumulation of ROS in the extracellular space [23]. On the other hand, alternative oxidase (AOX) may also have a role in the production of both ROS and RNS in the mitochondria [10,34,49], as it is a terminal oxidase of the mitochondrial electron transport chain, which can detoxify the OS and restore, to some extent, an equilibrium of their production [10,49].

Under unfavorable conditions, their generation may exceed the capacity of the plant to effectively control their concentration within the cell, so an accumulation of ROS and or RNS is inevitable. This elevated concentration causes oxidative damage, which is encountered at both cellular and molecular levels. To counteract oxidative stress, plants have evolved an array of defenses, including both enzymatic and non-enzymatic factors. Among the enzymatic ones, the major part is being played by superoxide dismutase (SOD), which dismutases O_2_^•–^ into H_2_O_2_, catalase (CAT), which detoxifies H_2_O_2_, peroxidase (POD), and enzymes of the ascorbate–glutathione cycle (such as ascorbate peroxidase (APX) and glutathione reductase (GR)) as well as other enzymes [1,3,45,50]. Among the non-enzymatic defense molecules against oxidative stress, phenolic compounds and ascorbic acid have a major role, along with sulfur-containing antioxidants such as glutathione, cysteine, tocopherols, and other antioxidant molecules [3,45,50,51,52].

At the physiological level, their role is quite important, since they serve as signal molecules and are involved in many developmental processes and plant morphogenesis. Among others, ROS take part in cell cycle and elongation, root hair formation, stomatal function, gravitropism, embryogenesis, lateral and adventitious root formation, root elongation, and other physiological processes [51,53,54].

It has been found that ROS may act downstream of several signaling pathways, which originate from the action of plant growth regulators [23,53]. Part of that action is the signal production for the formation of adventitious roots, especially considering that OS are actively produced after physical damage or wound, which is the starting point for the production of cuttings [23]. Nonetheless, there is not much known on the action of ROS or RNS in lateral as well as adventitious root formation; however, quite recently, enough research has been carried out to elucidate their role in this important physiological and economic process.

## 4. The Role and Function of Hydrogen Peroxide in Root Formation

**Figure 4:**
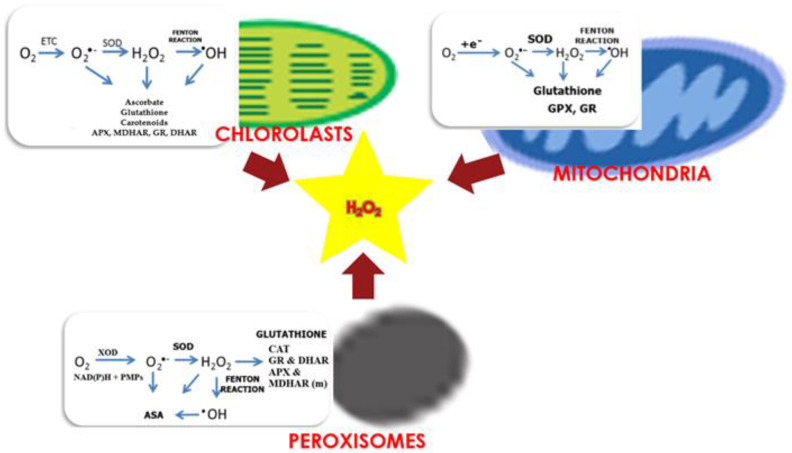
Production and scavenging of H_2_O_2_ in chloroplasts, mitochondria, and peroxisomes. ETC, electron transport chain; SOD, superoxide dismutase; DHAR, dehydroascorbate reductase; MDHAR, monodehydroascorbate reductase; CAT, catalase; POD, peroxidase; GPX, glutathione peroxidase; XOD, xanthine oxidase; PMPs, peroxisomal membranes polypeptides; GR, glutathione reductase; (m), indicates that APX and MDHAR are located in peroxisome membrane; ASA, ascorbic acid, (organelle figures were produced using EdrawMax software (Wondershare, Shenzhen, Guangdong, China) templates).

Hydrogen peroxide is a byproduct of aerobic metabolism [55] and is considered the most stable form of ROS, due to its relatively long life span (1 ms) [52,55]. It can be synthesized in peroxisomes, mitochondria, and chloroplasts (Figure 4) [3,55]. It is mainly produced via the dismutation of superoxide radical through the action of superoxide dismutase, while it can also be produced, directly or indirectly, by enzymes (cell-wall peroxidases, amine oxidases, and flavin-containing enzymes, glucose oxidases, glycolate oxidases, and sulfite oxidases) as well as through non-enzymatic processes [38,51,55]. It is a ubiquitous signal molecule, able to diffuse across membranes to different cellular compartments, participating in many physiological and metabolic processes of plants [52]. Furthermore, it has been found to use aquaporin channels to move across cells for signal transduction, thus spreading its impacts outside of the source cell [55]. At high concentrations, however, it can be toxic for the cells, enhancing lipid peroxidation and inducing programmed cell death and hypersensitive response (HR) [52,56]. Nonetheless, although it is a more oxidizing molecule than superoxide, it is biologically less toxic, as superoxide is toxic in picomolar intracellular concentrations, while cells can tolerate up to micromolar concentrations of H_2_O_2_. At non-toxic concentrations, it can serve as a signal molecule mediating systemic signal networks [38]. It also has a close relationship with some plant growth regulators, indirectly affecting plant physiological and molecular responses. It has been found to participate in the signaling pathways of cytokinins, jasmonic acid, indole-3-acetic acid, and ethylene [1,20]. At the same time, H_2_O_2_ is involved in the following processes:

- The regulation of plant defense arsenal against biotic and abiotic stress factors;
- Seed germination;
- The photosynthetic machinery;
- Cell viability and stomatal responses;
- The regulation of several proteins and the biosynthesis of amino acids;
- Cellular differentiation;
- Plant morphogenesis, and others [1,3,20,55].

Numerous studies have revealed the role of H_2_O_2_ in lateral root formation as well as in ARF in several species, both in woody perennials and annual plant species [1]. It has been found to induce root formation in olive cuttings in the presence of auxin, mung bean and cucumber hypocotyl cuttings, flax seedlings, Arabidopsis, marigold explants, peach–almond rootstock GF677, and ground cover chrysanthemum cuttings [53,57,58,59,60,61]. The positive effect of H_2_O_2_ on ARF seems to be dose- as well as species-dependent [3]. In olive, the application of H_2_O_2_ at 3.5% *w*/*v* plus the auxin indole-3-butyric acid (IBA) at 4000 ppm slightly increased the rooting percentage in various cultivars but improved the quality indexes of rooting, such as the number of roots [60]. In groundcover chrysanthemum cuttings, H_2_O_2_ at 200 μM proved to be more effective in inducing high rooting rates [58]. Similarly, in *Berberis thunbergii* var. *atropurpurea* cuttings, the application of H_2_O_2_ at 3.5% *w*/*v* in combination with IBA ta 3 g L^−1^ resulted in a higher rooting percentage than either control or solely IBA application [62]. On the other hand, the combined treatment of auxin plus H_2_O_2_ failed to induce any rooting in *Berberis vulgaris* var. *asperma* cuttings, indicating that the genotype may affect the rooting response [62]. H_2_O_2_ improved rooting quality traits in *Bougainvillea spectabilis* when combined with IBA at 1000 and 2000 mg L^−^^1^, without any significant effect on the rooting percentage [63]. Kordzadeh and Sarikhani [64] worked with the peach–almond rootstock GF677 stem cuttings, using different concentrations of auxin and H_2_O_2_ (IBA at 0, 1000, 2000, and 3000 mg L^−^^1^; and H_2_O_2_ at 1.5, 3 and 6% *w*/*v*) in three cutting seasons. Only in one season did the combined application of auxin (2000 mg L^−1^) plus H_2_O_2_ (3% *w*/*v*) prove to significantly improve the rooting performance of the cuttings. The combined application of IBA at 1000 mg L^−^^1^ plus H_2_O_2_ at 1.7% *w*/*v* increased the rooting percentages of pomegranate cv. Wonferful cuttings as well [65]. Similarly, the successive application of H_2_O_2_ at 50 mM plus IBA at 300 mg L^−^^1^ as a long pulse treatment resulted in increased rooting percentages in *Cordia trichotoma* (Vell.) Arrab. Ex Steud mini-cuttings [66]. Furthermore, the application of 20–40 mM H_2_O_2_ significantly increased the number of roots in cucumber seedling explants, while H_2_O_2_ up to 50 mM resulted in increased root fresh weight [51].

It has been reported, however, that H_2_O_2_ could have deleterious effects on adventitious rooting when applied alone without auxins [54], but this is not a common trait, since exogenous H_2_O_2_ has been found to mimic the auxin-induced adventitious rooting in cucumber and other species [53,59,67,68]. On the other hand, at high applied auxin concentrations, H_2_O_2_ inhibited root formation, indicating that overall, there is a balanced homeostasis between auxin and H_2_O_2_, which is necessary for the progress of adventitious rooting [54]. Taking into account that auxin has long been connected with adventitious root formation, even in the absence of H_2_O_2_ exogenous application, and that in some species, H_2_O_2_ presents synergistic effects with auxins in inducing higher rates of ARF, it can be assumed that H_2_O_2_ could probably upgrade the necessary auxin signaling for increased or improved ARF.

ROS in general, and more specifically H_2_O_2_, are closely connected to the wound response of cuttings, as it is produced through the action of diamine oxidases [3,6,20,56]. It has been found that its concentration increases within the first 12 h, reaching a maximum within 36 h after cutting severance from the mother plant [69,70], and this has been connected with the external application of auxin, suggesting the existence of feedback loops between auxin and peroxide [70]. This is further supported by the decreased activities of both POD and APX after auxin application [9], two enzymes responsible for the inactivation of H_2_O_2_ [71].

IBA application in mung bean cuttings has been found to increase H_2_O_2_ endogenous levels, suggesting that auxin treatment may enhance rooting through a pathway involving H_2_O_2_ [57] as well as polyamines, whose oxidation by polyamine oxidases leads to the production of H_2_O_2_ [34]. This also suggests a cross-talk between auxin and ROS signaling [3]. In olive cuttings, the basal external application of putrescine leads to increased rooting response, which is attributed to the increased POD level [34]. This in turn is the result of the enhanced putrescine degradation through the Δ’-pyrroline pathway, which generates H_2_O_2_, thus triggering the increase in POD activity and ultimately root response [28,34].

H_2_O_2_ has also been recognized as a downstream messenger of auxin signal distributed in the developing root primordia [1,53]. It has been found to mediate auxin signaling and possibly modify adventitious rooting via the cGMP and MAPK cascades [6,69]. The auxin-induced degradation of Aux/IAA repressors, which is necessary for ARF, is thought to be mediated via the cGMP stimulation induced by H_2_O_2_ [6]. According to Druege et al. [6], the accumulation of IAA through the PAT at the base of the cuttings in the future rooting zone can trigger a series of changes in auxin transport, signaling, and function, in those cells competent to dedifferentiate. These changes lead to a self-regulatory auxin accumulation, necessary to trigger ARF, while later on when auxin levels need to be reduced, this is accomplished by either catabolism (through the IAA oxidase action) and/or conjugation (triggered by Gretchen Hagen 3 (GH3) proteins) [6]. All these metabolic changes are under the orchestrated collaboration and fine-tuning of TIR1/AFB (transport inhibitor response 1/auxin signaling F-box) components, AUX/IAA proteins, and TPL (TOPLESS) co-repressor which guide the tissues through the different stages of ARF [6]. H_2_O_2_ can act as an auxin signaling mediator in order to coordinate the interaction of TIR1/AFB-Aux/IAA-ARFs (auxin response factors (ARFs)), through the cGMP and MAPK cascades, revealing its great mediating role [6].

Furthermore, in marigold plants, H_2_O_2_ application stimulated the endogenous Ca^2+^ and calmodulin (CaM) levels, which were closely related to the enhanced rooting response and acted as the downstream signal molecules of H_2_O_2_-induced adventitious rooting [33]. It has also been found to increase the activity of PPO, POD, and IAA oxidase, enzymes that are involved in the regulation of phenolic compounds and IAA, as well as cell redox homeostasis [32,62]. Taking into account that phenolic compounds may have a significant role in ARF, acting either as synergists or even inhibitors of rooting [24,40], it can be assumed that H_2_O_2_ has another collateral effect on ARF through the indirect regulation of rooting co-factors [44].

Moreover, it has been shown that the application of effective H_2_O_2_ scavengers or reducers, such as ascorbic acid as well as CAT [71], can eliminate the efficacy of exogenous H_2_O_2_ on ARF or even inhibit it when applied at high doses [37,51,55]. Similarly, when diphenylene iodonium (DPI), a strong inhibitor of NADPH oxidase, which is among the main sources of H_2_O_2_ formation, was applied in cucumber and mung bean seedlings, it reduced the formation and development of adventitious roots [37,51,69,70]. Thus, H_2_O_2_ scavengers or biosynthesis inhibitors could eliminate or partially inhibit the positive effects of H_2_O_2_ on ARF, strengthening, even more, its role in ARF.

Many genes are either up- or downregulated via H_2_O_2_ application. H_2_O_2_ increased the number of genes expressed during ARF compared with either water (control treatment) or IBA [1]. It upregulated the genes involved in transcription, DNA synthesis, cell cycle, hydrolase activity, cell-wall-related expansins, polygalacturonase genes, response to stress, oxidoreductase activity, isoflavonoid biosynthesis, sugar transporter genes, Gretchen Hagen 3 genes, and ethylene biosynthesis and downregulated the genes associated with oxidoreductase, Aux/IAA and ARF, cellular respiration, lipid transport, oxidative phosphorylation, lipoxygenase (LOX), cell-wall organization, and cellular biosynthetic process [1]. A common trend between IBA and H_2_O_2_-treated mung bean hypocotyl cuttings was the upregulation of genes involved in cell-wall loosening and the downregulation of the genes associated with cell-wall organization [1], indicating the necessary common pathway for cell-wall remodeling and the formation of new cells to participate in the root initials to be formed. Compared with the control treatment, an array of genes were upregulated, such as those controlling the pathways of glutathione metabolism and amino acid biosynthesis, purine metabolism, as well as cutin, suberin, and wax biosynthesis, starch and sugar metabolism, and protein processing [1]. It can be assumed that H_2_O_2_ triggered all the necessary actions for the first stages after cutting severance, aiming at fast wound healing, efficient stress response, carbohydrate accumulation, and energy control at the hypocotyl base. The increase in the total soluble sugar levels may be an indication of an enhanced signaling pathway to regulate cell division and differentiation, before the formation of root primordia [3]. On the other hand, it resulted in the downregulation of the genes involved in oxidative phosphorylation, phenylalanine metabolism, flavonoid and phenylpropanoid biosynthesis, and plant hormone signal transduction, as well as the AUX/IAA gene, thus initiating the auxin signaling leading to root initiation [1]. At the root initiation stage, H_2_O_2_ treatment resulted in an elevated expression of genes involved in stress responses, thus enhancing the cutting’s tolerance to stress [1]. Furthermore, H_2_O_2_ has been found to modulate (mostly increase) ethylene gene expression while repressing cytokinin- and gibberellin-related gene expression [1]. Taking into account that both cytokinins and gibberellins have been found to negatively affect rooting responses [12], it seems that H_2_O_2_ was able to reduce any such effect through the downregulation of their respective biosynthesis genes.

In summary, the external auxin application initiates and elevates the processes involved in auxin signaling, while H_2_O_2_ enhances those sequences responsible for stress tolerance and secondly those related to cell redox homeostasis, secondary metabolism, and cell-wall loosening. Through this induction, H_2_O_2_ can partly regulate the fate of ARF playing different roles during the various stages of adventitious rooting.

## 5. The Role and Function of Nitric Oxide in Root Formation

Nitric oxide (NO) is a diatomic free radical, potentially toxic, and relatively unstable gaseous molecule with multiple roles in signal transduction, regulating a plethora of physiological and biochemical processes in plants [35,48,55,70]. NO can react with various intracellular and extracellular molecules, forming a variety of products, such as NO radicals (NO^−^), nitrosonium ions (NO^+^), peroxynitrite (ONOO^−^), S-nitrosothiols (SNOs), higher oxides of nitrogen (NO_x_), and dinitrosyl iron complexes, which are all known as reactive nitrogen species (RNS) [48,53].

Nitrate reductase (NR), nitrite–NO reductase (Ni-NOR), xanthine oxidase (XOD), and NO synthase (NOS)-like enzymes are the enzymes responsible for the production of NO in plants [55]. It can also be produced by other non-enzymatic sources through nitrification or de-nitrification processes [55].

Generally, NO covalently attaches to the thiol side chain of cysteine (S-nitrosation) in proteins, resulting in the formation of S-nitrosothiols, and through this bonding, it regulates the transcriptional and post-translational processes involved in plant development [6,48,70,71]. The application of S-nitrosoglutathione (GSNO) in cucumber resulted in increased NO levels with a simultaneous decrease in S-nitrosoglutathione reductase (GSNOR) activity, which altogether led to an increase in ARF [71]. This provides further evidence that S-nitrosation might be involved in the NO-induced adventitious rooting, which is a NO-dependent post-translational modification (PTM) [71]. At the same time, auxin signaling is enhanced via S-nitrosation of the auxin receptor protein TIR1, thus facilitating the degradation of Aux/IAA [6] and through this the possible ARF. Besides all these, a NO-induced increase in IAA oxidase, POD, and PPO activities has been reported in marigold plants [32], which all lead to the conclusion that the NO-induced enhanced rooting is based on the multifunctional role of NO.

It can interact with various endogenous molecules, including plant hormones (auxin, cytokinin, and ethylene), during the early stages of growth and development [32,72]. It also interacts with other signaling molecules such as H_2_O_2_, salicylic acid, and jasmonic acid [6,48,72]. It is involved in every aspect of plant growth and development:

- Hypocotyl elongation;
- Seed germination and seedling development;
- Wounding;
- Abiotic stress tolerance;
- Senescence;
- Protection against pathogens;
- Dormancy release;
- Vegetative and reproductive growth;
- Seed germination;
- Leaf greening;
- Flower development and flowering time as well as pollen tube growth;
- Nutrient deficiency;
- Photosynthesis;
- Root organogenesis, root tip elongation, and lateral and adventitious root formation [32,35,46].

NO also triggers hypersensitive cell death and activates the expression of several defense genes [53,56]. It interacts with the plant hormone ethylene, another gaseous active molecule affecting the growth response of various tissues and organs [6,32]. At the same time, in coordination with ethylene, they can both alleviate the stress symptoms imposed by salinity and UV light [32], while as a control signal, NO is involved in several other stress responses such as drought, extreme temperature, and flooding anoxia [46,53]. Due to its many different actions, it has been suggested as a phytohormone with different properties than the traditional plant hormones [53].

It is reported that NO induces lateral root formation through the modulation of auxin homeostasis and cross-talk with ethylene and with other hormones and plant growth-regulating compounds [70].

At the same time, the wounding induced at the base of the cutting due to its severance from the mother plant enhances the activity of NR, resulting in increased NO production [43]. There is increasing evidence in recent years strengthening the role of NO in adventitious root formation, as it has been shown to promote rooting in Arabidopsis, cucumber, mung bean, marigold, *Eucalyptus grandis*, chrysanthemum and other plant species [33,58,69,73,74,75]. It was also found to be involved in the conversion of the applied IBA to IAA through the β-oxidation pathway and to increase the auxin binding to its receptors, favoring the formation of adventitious roots [76].

Furthermore, it is known that the cuttings derived from juvenile plant form roots easier than cuttings from mature ones [16,17]. It was found that NO accumulation is impaired in *Eucalyptus grandis* cuttings from mature mother plants, due to the decreased activity of NR, and this decrease was closely linked to reduced ARF [6]. The reported decrease in NR activity was attributed to the downregulation of a gene encoding NR in mature tissues. In fact, it was found that after the excision of the cutting from the mother plant, NO synthesis was greater in juvenile tissues than in mature ones [4,73].

The application of auxin at the cutting base has been linked to the increased production of NO. This production of NO in the sites of root formation seems to be critical, as it has been found to accumulate in cells, which divide and expand to form root primordia [77]. On the other hand, it has been proposed that auxin acts during the induction phase independently of NO formation, while initiation and expression phases require the interaction between IAA and NO [27]. Furthermore, the NO-induced stimulation of AR production may probably involve the strengthening of auxin signaling via the TIR1/AFB-Aux/IAA-ARFs interaction [6].

Cytokinins, on the other hand, are known to inhibit ARF [12,22,43]. According to Tailor et al. [43], cytokinins may downregulate the action of NO, thus suppressing its positive impact on ARF and in the end the cutting’s rhizogenesis.

Furthermore, the ethylene and NO interaction seems to induce ARF while at the same time improving the rooting quality indexes (root number and length). External ethylene treatment enhanced the endogenous levels of NO (possibly through the increased NOS and NR activities) as well as the development of adventitious roots in marigold plants [32,53], indicating that there may be a connection between ethylene and NO production on ARF and that NO is essential for the ethylene-induced ARF. Moreover, just as ethylene induces the formation of NO, NO has been reported to induce ethylene production, revealing a cross-talk between the two molecules [32,78]. Nonetheless, there are also reports on an antagonistic relationship between these two gases [32,78], which obscures their relationship.

The endogenous levels of NO are affected not only by auxins, cytokinins, and ethylene but also by jasmonates, while NO itself may also affect their concentration [74], supporting the cross-talk between NO and jasmonates. The peroxisomal cis-(+)-12-oxo-phytodienoic acid reductase (OPR3) activity (the enzyme involved in jasmonic acid biosynthesis) [79] is known to be enhanced by NO, revealing their synergistic effect. As jasmonates accumulate after cutting excision from the mother plant [23], it can be assumed that the relationship between jasmonates and NO may affect the outcome of ARF in cuttings as well. Another possible plant growth-regulating compound, melatonin, which is an indolamine sharing a common precursor with the natural auxin IAA, i.e., the amino acid tryptophan, has been found to promote ARF in several plants [65,80,81]. Melatonin seems to be implicated in auxin and NO signaling pathways, resulting in elevated concentration of both while at the same time enhancing the rooting potential and even more strengthening the role of NO in ARF [43].

There is also evidence that NO may activate two different pathways during the induction of ARF, the cGMP-dependent and cGMP-independent pathways [6,53]. It is clear by now that NO acts as a downstream messenger of the auxin-induced ARF [53,72,79]. On the one hand, this signal passes through the guanylate cyclase (GC)-catalyzed synthesis of cGMP, while on the other hand, it passes through a cGMP-independent pathway, as a MAPK signaling cascade is activated, which promotes ARF as well [53,67].

NO application in cucumber explants has been found to mimic the effect of IAA on inducing de novo root formation [82] and induce adventitious rooting to the same extent as IAA in many herbaceous plants [83]. Furthermore, an increase in soluble sugar concentration after NO treatment has been reported [13], indicating that NO may also have an indirect effect on ARF, by supplying carbon skeletons and through the energy provided by the accumulated carbohydrates [40]. On the other hand, NO application in tea cuttings was not able to induce ARF [69], probably due to the absence of some other, prerequisite factors for a successful NO-induced rhizogenesis. These factors were postulated to be soluble sugars, and more specifically sucrose, which would be accumulated at the sites where root initials would have been formed [69].

Furthermore, NO has been found to trigger the accumulation of calcium ions in the cytosol (Ca^2+^cyt) (through the mobilization of intracellular pools) and CaM endogenous content, activating, in turn, the CDPKs and MAPKs, leading to increased AR [33,84], while Ca^2+^ chelators and CaM antagonists prevent NO-induced AR [33], indicating that they have important roles in root formation and may act as downstream messengers in NO-induced adventitious rooting [33]. Ca^2+^/CaM complex and the regulation of CDPK activity have been reported to be involved in NO-induced AR formation under osmotic stress [85,86]. Similar involvement of Ca^2+^ and CDPKs has also been described for IAA-stimulated AR development [85], indicating a similar signal action of IAA and NO on inducing ARF.

Working backward, it is worth noting that the application of the NO scavenger 2-4-carboxyphenyl-4,4,5,5-tetramethylimidazoline-1-oxyl-3-oxide (c-PTIO) caused a significant reduction in the number of adventitious roots of IAA-treated cucumber explants [77], suggesting that NO is a prerequisite in IAA-induced rhizogenesis [53,72]. The external application of c-PTIO or the NO synthase inhibitor NG-nitro-L-arginine methyl ester (L-NAME) not only inhibited the production and accumulation of NO but also the brassinolide-induced ARF in cucumber explants [72], suggesting that NO is an important factor to be present during the early stages of rhizogenesis [53,55,70]. Similarly, the application of NaN_3_, an NR inhibitor, also resulted in decreased ARF in marigold plants [53]. Both L-NAME and NaN_3_ reduced the SNP-induced root number and length, possibly through their effects on the roles of NO itself [53]. Furthermore, while cell-cycle regulation in the xylem pericycle may progress to the G2 phase before ARF, the rest of the cells remain in the G1 phase, with NO inducing the progression of the cell cycle from the G1 phase to the S phase [35]. On the other hand, the application of c-PTIO and L-NAME as well as that of NaN_3_ all repressed the progression of cells to the S phase, with the subsequent inhibition of ARF [35]. Thus, it seems that NO is also involved in the regulation of cell-cycle-related genes and cell-cycle progression during ARF.

It is interesting, however, to see if the external application of putative NO donors, such as SNP, nitrate, or arginine (through the L-arginine dependent NO synthase), would result in an increased auxin-induced ARF in the cuttings of woody perennials, which are considered difficult to root.

## 6. The Combined Effects of H_2_O_2_ and NO on Root Formation

**Figure 5:**
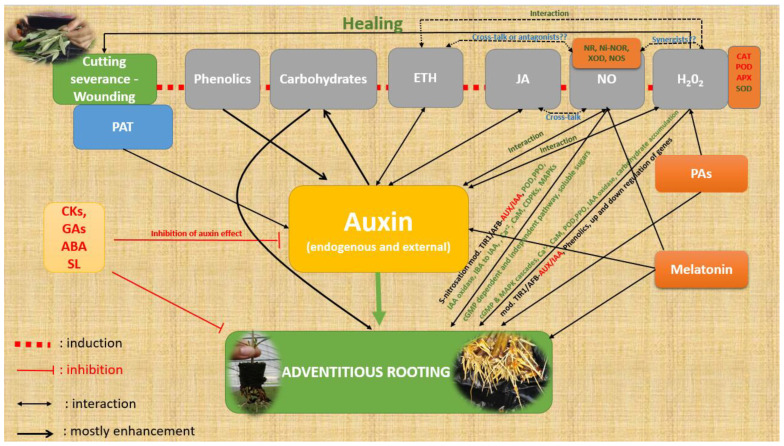
A simplified presentation of the factors described in this manuscript that influence ARF. Red letters indicate the catabolic activity of enzymes or reduction or inhibition, green letters indicate induction, activation, or increase, and black letters indicate modifications in general (increase/decrease). Interaction indicates the relationship between the specific compounds concerning ARF. ETH, ethylene; JA, jasmonates; NO, nitric oxide; NR, nitrate reductase; Ni-NOR, nitrite–NO reductase; XOD, xanthine oxidase; NOS, NO synthase-like enzymes; PAs, polyamines; CKs, cytokinins; Gas, gibberellins; ABA, abscisic acid; SL, strigolactones; PAT, polar auxin transport; POD, peroxidase; PPO, polyphenoloxidase; CAT, catalase; APX, ascorbate peroxidase; SOD, superoxide dismutase; IAA, indole-3-acetic acid; IBA, indole-3-butyric acid; CaM, calmodulin; cGMP, cyclic guanosine monophosphate; CDPKs, Ca^2+^-dependent protein kinases; MAPK, mitogen-activated protein kinase; TIR1/AFB (TRANSPORT INHIBITOR RESPONSE 1/AUXIN SIGNALING F-BOX).

Adventitious root formation is a developmental process involving multiple physiological, biochemical, and molecular alterations, which need to take place for the rooting stimulus to result in a successful root formation (Figure 5). Within this framework, the role of ROS and RNS is gradually elucidated, as described above. Nonetheless, it is not only their single effect that has a significant impact on ARF but also their combination and possible cross-talk [38,46,69]. They are both produced (simultaneously or in short succession of one another) upon wounding [53], while auxin treatment also triggers their production [6]; therefore, they could be considered key components of the molecular control of ARF. Their accumulation at the base of the cutting or the rooting zone in general depends on PAT as well [6]. They can both affect ARF by mediating auxin signaling, probably via the modification of the TIR1/AFB-Aux/IAA-ARF interaction, through the involvement of cGMP and MAPK cascades, leading to a gradual degradation of Aux/IAA repressors [6]. They both induce the accumulation of and increase in Ca^2+^ and CaM [33], which, as already stated above, have a role in ARF. Thus, they are both considered molecules acting downstream of auxin during the ARF [23]. It has been suggested that, as they both affect similar signaling pathways, they may exert additive or even synergistic responses [53]. When H_2_O_2_ and an NO donor were applied together in marigold plants, they enhanced the number and length of the produced adventitious roots compared with those produced by the single application of either H_2_O_2_ or NO alone [53], supporting their synergistic action. According to Kora and Bhattacharjee [3], H_2_O_2_ acts as a downstream signal of NO signaling during ARF. In wild-type *Arabidopsis thaliana*, the formation of adventitious roots by H_2_O_2_ is accomplished through the increased concentration of NO [87], possibly through the increased activity of NR [55], indicating a synergistic and cumulative rooting action of these two molecules. It has also been suggested that NO can trigger the production of H_2_O_2_ as well, as the application of either cPTIO or L-NAME inhibited the production of H_2_O_2_ and resulted in decreased ARF [55]. On the other hand, during the ARF in marigold plants, some root-inducing signaling molecules were exclusively attributed to NO signaling, while others specifically to H_2_O_2_ signaling, thus revealing both their differences and their common or parallel way of action and cross-talk [55].

## 7. Conclusions

Adventitious rooting is a well-orchestrated process, in which many molecules take part, and their action must be synchronized to achieve maximum results. Auxin, and plant growth regulators in general, surely play a critical role in the induction of adventitious rooting, but the role of new molecules has been increasingly highlighted in recent decades. According to the rhizocaline theory, which was developed at the beginning of the previous century, adventitious rooting progresses through the effect of auxin as well as a non-auxin stimulus that is a complex of biochemical factors, and together, both these stimuli control the rooting response. Among these factors, the role of ROS and RNS, and more specifically H_2_O_2_ and NO, has gradually been elucidated. However, more research is needed to fully understand their action, synergists, and complexes with other molecules, which may or may not affect ARF.

## Data Availability

Manuscripts used in this review are available upon request by the author.
